# Supplementary figures and images for: Sorafenib/2800Z Co-Loaded into Cholesterol and PEG Grafted Polylysine NPs for Liver Cancer Treatment
Source: Pharmaceuticals (Basel). 2023 Jan 13;16(1):119. doi: 10.3390/ph16010119 (PMC9865580; doi:10.3390/ph16010119)

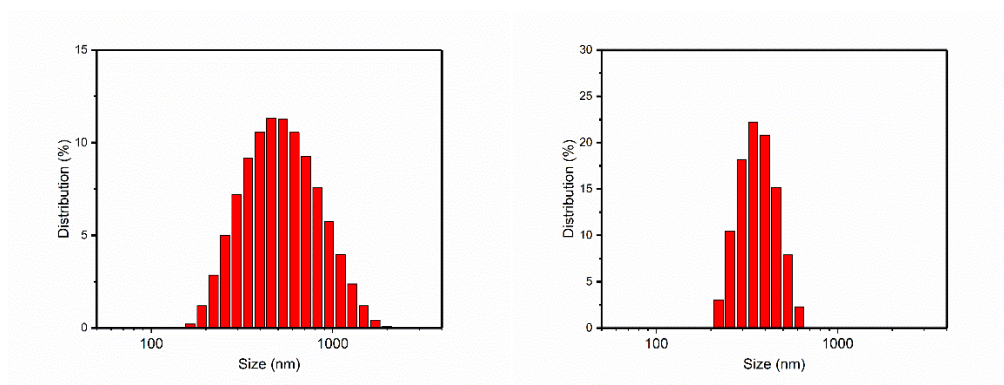

Figure S1 Hydrodynamic dimension of RED@PsPC (left,  $354.2 \pm 1.2$  nm) and ICG@PsPC (right,  $365.7 \pm 2.6$  nm).

Supplement: Supplementary file 1 [file pharmaceuticals-16-00119-s001.zip › pharmaceuticals-2082766-supplementary.pdf]
